# Diagnostic change 10 years after a first episode of psychosis

**DOI:** 10.1017/S0033291715000720

**Published:** 2015-05-04

**Authors:** M. Heslin, B. Lomas, J. M. Lappin, K. Donoghue, U. Reininghaus, A. Onyejiaka, T. Croudace, P. B. Jones, R. M. Murray, P. Fearon, P. Dazzan, C. Morgan, G. A. Doody

**Affiliations:** 1 Centre for Economics of Mental and Physical Health, King's College London, London, UK; 2 Division of Psychiatry, University of Nottingham, Nottingham, UK; 3 Department of Psychiatry, University of New South Wales, Sydney, Australia; 4 Psychosis Studies Department, King's College London, London, UK; 5 Addictions Department, King's College London, London, UK; 6 Centre for Epidemiology and Public Health, King's College London, London, UK; 7 Department of Psychiatry and Psychology, School for Mental Health and Neuroscience, Maastricht University, Maastricht, The Netherlands; 8 NIHR Collaboration for Leadership in Applied Health Research & Care, Cambridge, UK; 9 Department of Psychology, King's College London, London, UK; 10 School of Nursing and Midwifery, College of Medicine, Dentistry and Nursing, University of Dundee, Dundee, UK; 11 Department of Psychiatry, University of Cambridge, Cambridge, UK; 12 Department of Psychiatry, Trinity College, Dublin, Republic of Ireland

**Keywords:** Diagnosis, first-episode psychosis, psychoses

## Abstract

**Background:**

A lack of an aetiologically based nosology classification has contributed to instability in psychiatric diagnoses over time. This study aimed to examine the diagnostic stability of psychosis diagnoses using data from an incidence sample of psychosis cases, followed up after 10 years and to examine those baseline variables which were associated with diagnostic change.

**Method:**

Data were examined from the ÆSOP and ÆSOP-10 studies, an incidence and follow-up study, respectively, of a population-based cohort of first-episode psychosis cases from two sites. Diagnosis was assigned using ICD-10 and DSM-IV-TR. Diagnostic change was examined using prospective and retrospective consistency. Baseline variables associated with change were examined using logistic regression and likelihood ratio tests.

**Results:**

Slightly more (59.6%) cases had the same baseline and lifetime ICD-10 diagnosis compared with DSM-IV-TR (55.3%), but prospective and retrospective consistency was similar. Schizophrenia, psychotic bipolar disorder and drug-induced psychosis were more prospectively consistent than other diagnoses. A substantial number of cases with other diagnoses at baseline (ICD-10, *n* = 61; DSM-IV-TR, *n* = 76) were classified as having schizophrenia at 10 years. Many variables were associated with change to schizophrenia but few with overall change in diagnosis.

**Conclusions:**

Diagnoses other than schizophrenia should to be regarded as potentially provisional.

## Introduction

Diagnosis in psychiatry has frequently come under fire. Robins & Guze (1970) discussed how clinical features, outcome and family history can be used to create nosological categories in the absence of clinical tests. The National Institute of Mental Health recently criticized the validity of the Diagnostic and Statistical Manual of Mental Disorders (DSM), stating ‘Unlike our definitions of ischemic heart disease, lymphoma, or AIDS, the DSM diagnoses are based on a consensus about clusters of clinical symptoms, not any objective laboratory measure’ (Lane, 2013). This lack of aetiologically based psychiatric classification has contributed to instability in psychiatric diagnoses over time.

A number of studies have examined diagnostic stability over time. Early research was heterogeneous in nature, reporting on differing diagnostic criteria, different diagnostic processes and differing methods of reporting results. More recently, however, researchers have followed the lead of Schwartz *et al.* (2000) in reporting prospective and retrospective consistencies of diagnoses between two time points. Prospective consistency is the proportion of cases that receives a diagnosis at baseline and retains that diagnosis at follow-up. Retrospective consistency is the proportion of cases that receives a diagnosis at follow-up that they also had at baseline.

The prospective consistencies of schizophrenia and bipolar affective disorder have been reported at 80–100% in studies with up to 4 years of follow-up using first admission or first contact samples and more recent criteria [International Classification of Diseases (ICD)-10 or DSM-III onwards] (Amin *et al.* 1999; Schwartz *et al.* 2000; Veen *et al.* 2004; Baldwin *et al.* 2005; Whitty *et al.* 2005; Salvatore *et al.* 2009, 2011). Studies with shorter follow-up periods report that diagnoses are more stable over time. Longer-term follow-up of the cohorts, identified by the Determinants of Outcome of Severe Mental *Disorder* (DOSMeD), World Health Organization Collaborative Study on the Assessment and *Reduction of Social Disability* (RAPyD) and International Pilot Study of Schizophrenia (IPSS) samples reported by Craig *et al.* (2007) identified prospective consistencies for schizophrenia of about 85% over 12–26 years of follow-up, using ICD-10 diagnoses converted using a World Health Organization algorithm from previous classification systems. Bromet *et al.* (2011) reported on the 10-year follow-up of a first admission cohort and identified similar diagnostic stability findings for schizophrenia and bipolar affective disorder.

Findings with regards to other psychotic categories are more variable. Depressive psychosis prospective consistencies are reported to be between 45 and 55% (Baca-Garcia *et al.* 2007*a*, *b*; Bromet *et al.* 2011) in long-term follow-up studies (10–12 years), compared with 75–100% in shorter-term follow-up studies (1.5–4 years; Schwartz *et al.* 2000; Schimmelmann *et al.* 2005; Whitty *et al.* 2005; Salvatore *et al.* 2009, 2011). Smaller diagnostic categories (where studied) demonstrate more variation still. Schizo-affective disorder, for example, has reported prospective consistencies ranging from 35% (Schwartz *et al.* 2000; Craig *et al.* 2007) to 95–100% (Schimmelmann *et al.* 2005; Salvatore *et al.* 2011). This variation probably reflects differences in the diagnostic system adopted, cohort (first contact *v.* first hospitalization) and length of follow-up. Further studies have attempted to address stability in less prevalent diagnoses such as acute and transient psychosis. These studies also show considerable variability in prospective consistency: 30% (Aadamsoo *et al.* 2011); 73% (Sajith *et al.* 2002).

Many studies mentioned above used non-incidence samples. Incidence studies give a less biased estimate of diagnostic stability, as studies that recruit cases from non-first-episode samples are effectively sampling chronic cases in treatment, biasing the investigation towards those who are more unwell and excluding others who recover. One study has examined long-term diagnostic stability (13 years) in an incidence sample of psychosis patients but only including schizophrenia cases (Mason *et al.* 1997). To date, no study has yet examined diagnostic stability of all psychosis diagnoses specifically in incident cases over a follow-up period of longer than 8 years using current diagnostic criteria.

Identification of predictors of diagnostic change over time is important because predictors may aid in understanding why diagnoses change and indicate to clinical teams when to be attentive to potential change and adjust treatment accordingly. Studies of relatively short duration have examined predictors of diagnostic change in an incidence cohort, but only one study has looked at a long-term follow-up of diagnostic categories, in first admission cases (Schwartz *et al.* 2000; Salvatore *et al.* 2009; Bromet *et al.* 2011).

In this study we sought to complement the knowledge obtained from previous research, using a geographically defined incident sample of all psychoses diagnoses made using DSM-IV-TR and ICD-10 criteria, followed up and rediagnosed 10 years later, to fulfil two main aims: (1) to examine the stability of diagnostic categories 10 years after the first episode of psychosis; and (2) to examine which demographic and clinical characteristics are associated with diagnostic change.

## Method

### Baseline

This paper is based on data from the ÆSOP study (Kirkbride *et al.* 2006; Morgan *et al.* 2014), an incidence study of all first-episode psychosis cases presenting for the first time to specialist mental health services in defined catchment areas in Nottingham and London.

At baseline, clinical and demographic data were collected from clinical records and, where possible, from interview with cases using the Schedules for Clinical Assessment in Neuropsychiatry (SCAN version 2; World Health Organization, 1994) and the Personal and Psychiatric History Schedule (World Health Organization, 1996). The SCAN was used to elicit symptom-related data at the time of presentation. Where an interview with the patient was not possible, case notes were used to complete the Item Group Checklist (IGC) part of the SCAN. Case summaries collated all relevant clinical information and were made available to consensus diagnostic meetings alongside the schedules above. ICD-10 (World Health Organization, 1993) and DSM-IV-TR (American Psychiatric Association, 2000) psychotic diagnoses were determined using all available clinical information (excluding clinical diagnosis) on the basis of consensus meetings involving at least one of the principal investigators with other members of the research team (at least one psychiatrist and one other member of the research team). Conflicting opinions on diagnosis were discussed in detail until a consensus was reached. This was made as soon as possible after first contact (generally within a few weeks). Cases with a dual diagnosis had their substance use disorder diagnosed separately. Diagnoses were made blind to ethnicity. Factor analyses of the SCAN and IGC data generated the following symptom dimensions: manic; depressive; disorganized; negative; and reality distortion (Demjaha *et al.* 2009).

### Follow-up

Cases were followed up 10 years after first contact with services in ÆSOP-10 (Morgan *et al.* 2014). The World Health Organization Life Chart (Susser *et al.* 2000; Harrison *et al.* 2001) was completed for each case using case notes and clinical interview where possible, to map course of illness and symptom history. The SCAN was also completed in relation to the preceding month where possible. Lifetime diagnosis (based on information from baseline to follow-up) using a consensus approach was based on all this clinical information, and blind to ethnicity and baseline diagnoses.

Ethical approval was granted by the Institute of Psychiatry and South London and Maudsley (SLaM) Research Ethics Committee and by the North Nottingham Healthcare NHS Trust Ethics Committee.

### Analyses

Data were analysed using STATA 10 (StataCorp, 2009). Differences between followed-up and not-followed-up cases were analysed using χ^2^ and Wilcoxon rank-sum tests as appropriate. Prospective and retrospective consistencies were calculated as the percentage of cases with the same diagnosis at follow-up as at baseline, and the percentage of cases with the same diagnosis at baseline as at follow-up, respectively.

Predictors of diagnostic change were assessed in two steps: unadjusted univariate logistic regression analyses; and models of independent predictors. Models of independent predictors were built by starting with a single variable and adding in additional variables, one at a time to examine their effect on the model. Only variables with *p* < 0.1 in the unadjusted analyses were entered into the adjusted regression model starting with the variable with the biggest effect. Each additional variable was then added to the model in turn using likelihood ratio tests to determine if that variable significantly improved the model or not. Variables that did not improve the model were excluded from the model. Predictors were simplified into binary factors where possible in order to simplify analyses and increase power. As well as analysing age as a continuous variable, it was reclassified into a binary variable (in the age risk period: males under the age of 40 years or females under the age of 50 years) as this has been indicated as an important predictor in the development of schizophrenia (Häfner *et al.* 1998).

## Results

### Sample

At baseline, a total of 557 first-episode psychosis cases were identified. Data here are based on the incidence sample collected over the first 2 years (excluding: non-incidence cases collected for the brain imaging component of the study; cases oversampled in the third year in order to increase the numbers for the ethnicity component of the study; and cases excluded post-baseline). This led to a total number of 505 cases: 304 from London and 201 from Nottingham.

Of the 505 patients eligible for follow-up, 79.8% (403) had sufficient information to make a lifetime diagnosis based on at least 8 years of information (including 33 cases who had died during the follow-up period but had sufficient information to have a lifetime diagnosis made). Therefore, a total of 102 cases had no follow-up diagnostic information. The mean follow-up in years of those who had a lifetime diagnosis and were still alive was 10.74 years (s.d. = 1.17, range 8.08–13.70).

Table 1 shows the differences in demographic and clinical variables between those with and without a diagnosis at 8 or more years. Centre and place of birth were associated with lifetime diagnosis status. Cases who were born abroad were more likely to move abroad over the follow-up period than cases born in the UK and therefore less likely to be followed up. More cases from London were not born in the UK (38%, 112/297) compared with Nottingham (9%, 18/201; *p* < 0.01), and so a lower lifetime diagnosis rate in London could be linked to place of birth.

**Table 1.** Comparison of key variables between those with a diagnosis and those without a diagnosis

			Test statistic		
	No diagnosis, *n* (%) (*n* = 102)	Diagnosis, *n* (%) (*n* = 403)	*χ*^2^ (df)	Wilcoxon *Z*	*p*
Baseline diagnosis					
Schizophrenia	41 (18.8)	177 (81.2)	2.088 (8)		0.973 (Fisher's exact)
Delusional disorder	3 (13.6)	19 (86.4)			
Acute and transient psychoses	7 (24.1)	22 (75.9)			
Schizo-affective	6 (19.4)	25 (80.7)			
Bipolar disorder and mania with psychotic features	15 (21.4)	55 (78.6)			
Major depression with psychotic symptoms	17 (23.6)	55 (76.4)			
Schizotypal personality disorder	0 (−)	1 (100)			
Drug-induced psychoses	5 (19.2)	21 (80.8)			
Psychoses NOS	8 (22.2)	28 (77.8)			
Grouped diagnoses					
Non-affective psychoses	70 (19.2)	293 (80.7)	0.7744 (2)		0.679
Bipolar disorder and mania with psychotic features	15 (21.4)	55 (78.6)			
Major depression with psychotic symptoms	17 (23.6)	55 (76.4)			
Centre					
London	73 (24.0)	231 (76.0)	6.897 (1)		0.009
Nottingham	29 (14.4)	172 (85.6)			
Gender					
Male	63 (21.5)	230 (78.5)	0.736 (1)		0.391
Female	39 (18.4)	173 (81.6)			
Ethnicity					
White British	43 (18.9)	185 (81.1)	10.570 (5)		0.061
African-Caribbean	18 (15.1)	101 (84.9)			
Black African	17 (26.2)	48 (73.9)			
White other	10 (27.8)	26 (72.2)			
Asian	3 (11.5)	23 (88.5)			
Other	11 (35.5)	20 (64.5)			
Place of birth					
Non-UK born	35 (26.9)	95 (73.1)	4.800 (1)		0.028
UK born	66 (17.9)	302 (82.1)			
Age at first contact, years				−1.617	0.106
Median	27	29			
IQR	22–33	22–36			
Age at onset, years				−1.597	0.110
Median	27	29.5			
IQR	22–34	23–38			
DUP, days				−0.248	0.804
Median	56	60			
IQR	15–184	15–238			

df, Degrees of freedom; NOS, not otherwise specified; IQR, interquartile range; DUP, duration of undiagnosed psychosis.

### Diagnostic change: prospective and retrospective consistency

Of cases, 59.6% (240/403) had the same baseline and lifetime ICD diagnosis, and 55.3% (223/403) of cases had the same baseline and lifetime DSM diagnosis. A substantial proportion of patients had a baseline and lifetime diagnosis of schizophrenia in both the ICD (33%) and DSM (28%) (Tables 2 and 3). The DSM system produced four cases with a baseline or lifetime diagnosis of either ‘unknown’ or ‘other’. This was due to the fact that although there was a large amount of information for 8 or more years, the diagnostic team was unable to agree upon a diagnosis.

**Table 2.** ICD movement matrix

	Baseline diagnosis (*n*)									
Lifetime diagnosis (*n*)	Schizophrenia	Schizo-affective disorder	Delusional disorder	Acute and transient psychoses	Bipolar disorder and mania with psychotic features	Major depression with psychotic features	Schizotypal personality disorder	Drug-induced psychoses	Psychosis NOS	Total
Schizophrenia	133	8	12	7	2	15	1	3	13	194
Schizo-affective disorder	16	*9*	0	0	3	3	0	1	5	37
Delusional disorder	0	0	*3*	1	0	2	0	0	0	6
Acute and transient psychoses	1	1	0	4	2	1	0	1	0	10
Bipolar disorder and mania with psychotic features	7	3	0	1	42	7	0	0	1	61
Major depression with psychotic features	8	1	1	5	2	26	0	0	1	44
Schizotypal personality disorder	0	0	0	0	0	0	*0*	0	1	1
Drug-induced psychoses	5	1	0	5	2	0	0	*16*	0	29
Psychoses NOS	7	2	1	1	2	1	0	0	*7*	21
Total	177	25	17	24	55	55	1	21	28	403

ICD, International Classification of Diseases; NOS, not otherwise specified.

**Table 3.** DSM movement matrix

	Baseline diagnosis (*n*)												
Lifetime diagnosis (*n*)	Schizophrenia	Schizo-affective disorder	Major depression with psychotic features	Bipolar disorder and mania with psychotic features	Delusional disorder	Psychosis NOS	Schizophreniform	Drug/alcohol-induced psychosis	Other disorder	Unclear	Brief psychotic disorder	Total
Schizophrenia	113	8	15	2	12	13	17	4	1	1	3	189
Schizo-affective disorder	13	6	4	3	0	5	1	0	0	0	0	32
Major depression with psychotic features	7	1	28	2	1	1	1	0	0	0	3	44
Bipolar disorder and mania with psychotic features	7	2	9	42	0	1	0	0	0	0	1	62
Delusional disorder	0	0	2	0	4	0	0	0	0	0	0	6
Psychosis NOS	6	3	1	3	1	8	1	0	0	0	1	24
Schizophreniform	3	0	0	0	1	1	3	0	0	0	0	8
Drug / alcohol induced psychosis	4	1	0	2	2	1	2	16	0	0	1	29
Other disorder	1	0	0	0	0	0	0	0	*0*	0	0	1
Unclear	0	0	0	0	0	0	0	0	0	*0*	1	1
Brief psychotic disorder	1	0	1	1	0	0	0	1	0	0	*3*	7
Total	155	21	60	55	21	30	25	21	1	1	13	403

DSM, Diagnostic and Statistical Manual of Mental Disorders; NOS, not otherwise specified.

Table 4 presents the prospective and retrospective consistency of the ICD-10 and DSM-IV-TR. The DSM and ICD had very similar prospective consistencies for most diagnoses, with schizophrenia, psychotic bipolar disorder and drug-induced psychosis having the highest prospective consistency. In terms of retrospective consistency, schizophrenia diagnosed using the DSM had almost 10% lower consistency than schizophrenia diagnosed using the ICD. This difference is accounted for by the use of schizophreniform disorder in the DSM but not in the ICD. Drug-induced psychosis had a marginally higher prospective consistency compared with schizophrenia when using both the ICD and DSM. There were more cases at follow-up than at baseline with the following diagnoses: schizophrenia, schizo-affective disorder, bipolar disorder and drug-induced psychosis.

**Table 4.** Prospective and retrospective consistency

	Prospective consistency, % (baseline number)		Retrospective consistency, % (follow-up number)	
Diagnosis	DSM	ICD	DSM	ICD
Schizophrenia	72.9 (155)	75.1 (177)	59.8 (189)	68.6 (194)
Bipolar disorder and mania with psychotic features	76.4 (55)	76.4 (55)	67.7 (62)	68.9 (61)
Major depression with psychotic features	46.7 (60)	47.3 (55)	63.6 (44)	59.1 (44)
Schizo-affective disorder	28.6 (21)	36.0 (25)	18.8 (32)	24.3 (37)
Delusional disorder	19.0 (21)	17.6 (17)	66.7 (6)	50.0 (6)
Drug-induced psychoses	76.2 (21)	76.2 (21)	55.2 (29)	55.2 (29)
Psychoses NOS	26.7 (30)	25.0 (28)	33.3 (24)	33.3 (21)
Schizophreniform	12.0 (25)	–	37.5 (8)	–
Brief/acute and transient psychoses	23.1 (13)	16.7 (24)	37.5 (7)	40.0 (10)

DSM, Diagnostic and Statistical Manual of Mental Disorders; ICD, International Classification of Diseases; NOS, not otherwise specified.

### Predictors of change

The unadjusted regression analyses for key baseline demographic, clinical and social variables revealed that five baseline variables were associated with change in ICD diagnosis (see online Supplementary Appendix S1 for full details). Likelihood ratio tests revealed that the variables which together created the most parsimonious model associated with change were: having a diagnosis of delusional disorder [odds ratio (OR) 23.42, 95% confidence interval (CI) 4.15–132.03], acute and transient psychosis (OR 73.84, 95% CI 8.52–639.80), schizo-affective disorder (OR 9.00, 95% CI 2.33–34.71) or psychosis not otherwise specified (NOS) (OR 12.74, 95% CI 2.24–72.39); being from London (Nottingham: OR 0.50, 95% CI 0.25–1.04); and having depressive symptoms (OR 1.92, 95% CI 1.11–3.32).

Eight baseline variables were associated with change in DSM diagnosis in the unadjusted analyses. Likelihood ratio tests revealed that the variables which together created the most parsimonious model of change were: having a diagnosis of psychotic major depression (OR 2.54, 95% CI 0.87–7.41), schizo-affective disorder (OR 12.21, 95% CI 2.16–69.00), delusional disorder (OR 40.02, 95% CI 6.39–250.73), psychosis NOS (OR 22.95, 95% CI 3.88–135.76) and brief psychotic disorder (OR 39.38, 95% CI 3.53–439.13); being from London (Nottingham: OR 0.46, 95% CI 0.22–0.98); having a lower age at onset (OR 0.17, 95% CI 0.05–0.54); having contact with friends (less than weekly contact with friends: OR 0.34, 95% CI 0.13–0.90); and having depressive symptoms (OR 2.35, 95% CI 1.30–4.26).

### Predictors of change to schizophrenia

Considering the high number of cases moving to a diagnosis of schizophrenia over the follow-up period and the implied increase in need this may engender (Carr *et al*. 2004), a separate analysis of predictors of change to a diagnosis of schizophrenia was undertaken. This is shown in Table 5. For the ICD analyses, all diagnoses were used. However, given the overlap between DSM-IV-TR schizophreniform psychosis and schizophrenia, schizophreniform cases were excluded from the DSM analysis.

**Table 5.** Predictors of diagnostic change to schizophrenia using the ICD and DSM

Predictor	ICD unadjusted OR (95% CI)	Predictor	DSM unadjusted OR (95% CI)
Demographics			
Centre (*n* = 226)		Centre (*n* = 223)	
London	–	London	–
Nottingham	0.31 (0.16–0.58)**	Nottingham	0.34 (0.18–0.65)**
Gender (*n* = 226)		Gender (*n* = 223)	
Male	–	Male	–
Female	0.57 (0.31–1.03)*	Female	0.50 (0.27–0.92)**
Log age (*n* = 226)	0.62 (0.25–1.50)	Log age (*n* = 223)	0.49 (0.20–1.23)
In the age risk period (*n* = 226)		In the age risk period (*n* = 223)	
No	–	No	–
Yes	3.43 (1.16–10.14)**	Yes	3.46 (1.17–10.24)**
Ethnicity (*n* = 226)		Ethnicity (*n* = 223)	
White British	–	White British	–
African-Caribbean	1.50 (0.70–3.22)	African-Caribbean	1.32 (0.61–2.87)
Black African	3.03 (1.25–7.35)**	Black African	2.77 (1.15–6.65)**
White other	2.36 (0.62–8.99)	White other	2.31 (0.60–8.80)
Asian	0.97 (0.25–3.72)	Asian	1.04 (0.27–4.05)
Other	0.79 (0.16–3.87)	Other	0.38 (0.05–3.18)
Clinical			
Diagnosis (*n* = 225)		Diagnosis (*n* = 221)	
Psychoses NOS	–	Brief psychotic disorder	–
Delusional disorder	1.98 (0.60–6.51)	Major depression with psychotic features	1.11 (0.27–4.58)
Acute and transient psychoses	0.54 (0.17–1.73)	Bipolar disorder and mania with psychotic features	0.13 (0.02–0.85)**
Schizo-affective	0.54 (0.18–1.67)	Schizo-affective	2.05 (0.43–9.78)
Bipolar disorder and mania with psychotic features	0.04 (0.01–0.21)**	Delusional disorder	4.44 (0.94–21.00)*
Major depression with psychotic features	0.43 (0.17–1.12)*	Psychoses NOS	2.55 (0.58–11.18)
Schizotypal personality disorder	–	Drug-induced psychoses	0.78 (0.14–4.24)
Drug-induced psychoses	0.19 (0.05–0.80)**	Other disorder	–
		Unknown/unclear	–
Family history of psychosis (*n* = 158)		Family history of psychosis (*n* = 157)	
No	–	No	–
Yes	1.07 (0.47–2.46)	Yes	1.39 (0.61–3.16)
Log DUP, days (*n* = 219)	1.16 (0.99–1.35)*	Log DUP, days (*n* = 216)	1.14 (0.98–1.33)*
Log age of onset (*n* = 219)	0.65 (0.27–1.57)	Log age of onset (*n* = 216)	0.51 (0.21–1.27)
Any drug use at baseline (*n* = 207)		Any drug use at baseline (*n* = 204)	
No use	–	No use	–
Use	0.83 (0.45–1.56)	Use	0.97 (0.52–1.82)
Mode of onset (*n* = 200)		Mode of onset (*n* = 195)	
Sudden	–	Sudden	–
Acute	0.63 (0.25–1.57)	Acute	0.56 (0.22–1.42)
Insidious	1.32 (0.61–2.86)	Insidious	1.22 (0.56–2.66)
Symptom dimension–log (*n* = 202) mania	0.35 (0.21–0.57)**	Symptom dimension–log (*n* = 201) mania	0.37 (0.23–0.60)**
Symptom dimension–log (*n* = 202) reality distortion	1.73 (1.07–2.81)**	Symptom dimension–log (*n* = 201) reality distortion	1.77 (1.08–2.91)**
Symptom dimension–log (*n* = 202) negative	1.84 (1.15–2.93)**	Symptom dimension–log (*n* = 201) negative	1.77 (1.10–2.87)**
Symptom dimension–log (*n* = 202) depression	1.03 (0.68–1.56)	Symptom dimension–log (*n* = 201) depression	1.09 (0.72–1.66)
Symptom dimension–log (*n* = 202) disorganization	0.73 (0.37–1.46)	Symptom dimension–log (*n* = 201) disorganization	0.70 (0.35–1.41)
Social			
Living situation (*n* = 223)		Living situation (*n* = 220)	
Alone	–	Alone	–
Not alone	0.49 (0.26–0.89)**	Not alone	0.47 (0.25–0.87)**
Relationship status (*n* = 218)		Relationship status (*n* = 215)	
Single	–	Single	–
Not single	0.27 (0.12–0.59)**	Not single	0.28 (0.13–0.61)**
Highest education level (*n* = 220)		Highest education level (*n* = 218)	
School	–	School	–
Further	0.52 (0.25–1.11)*	Further	0.46 (0.21–0.99)**
Higher	0.61 (0.25–1.53)	Higher	0.60 (0.24–1.50)
Employment status (*n* = 218)		Employment status (*n* = 215)	
Unemployed	–	Unemployed	–
Other	0.55 (0.28–1.06)*	Other	0.64 (0.33–1.22)
Contact with friends (*n* = 148)		Contact with friends (*n* = 147)	
Daily	–	Daily	–
Weekly	2.24 (0.76–6.56)	Weekly	1.88 (0.67–5.28)
Less than weekly	4.49 (1.54–13.05)**	Less than weekly	3.50 (1.24–9.85)**

ICD, International Classification of Diseases; DSM, Diagnostic and Statistical Manual of Mental Disorders; OR, odds ratio; CI, confidence interval; NOS, not otherwise specified; DUP, duration of undiagnosed psychosis.

* *p* < 0.1, ** *p* < 0.05.

The unadjusted analyses revealed that many more variables were associated with ICD and DSM diagnosis change to schizophrenia. For the ICD, there was some evidence that 14 baseline variables were associated with a change to schizophrenia. There was strong evidence that the following were associated with change to schizophrenia: being from London (Nottingham OR 0.31, 95% CI 0.16–0.58); being in the age risk period (OR 3.43, 95% CI 1.16–10.14); being black African (OR 3.03, 95% CI 1.25–7.35); longer duration of undiagnosed psychosis (DUP) (OR 1.16, 95% CI 0.99–1.35); having symptoms of reality distortion (OR 1.73, 95% CI 1.07–2.81); having negative symptoms (OR 1.84, 95% CI 1.15–2.93); living along (not living alone OR 0.49, 95% CI 0.26–0.89); being single (not single OR 0.27, 95% CI 0.12–0.59); and having contact with friends less than weekly (OR 4.49, 95% CI 1.54–13.05).

There was strong evidence that the following baseline variables were associated with not changing diagnosis to schizophrenia: having a diagnosis of bipolar disorder (OR 0.04, 95% CI 0.01–0.21) or drug-induced psychoses (OR 0.19, 95% CI 0.05–0.80); and having manic symptoms (OR 0.35, 95% CI 0.21–0.57).

There was some weak evidence that the following were associated with changing to a diagnosis of schizophrenia: being male (female OR 0.57, 95% CI 0.31–1.03); having psychotic major depression (OR 0.43, 95% CI 0.17–1.12); being unemployed (other OR 0.55, 95% CI 0.28–1.06); and not being in further education (further education OR 0.52, 95% CI 0.25–1.11).

For the DSM, there was some evidence that 13 baseline variables were associated with a change to schizophrenia. There was strong evidence that the following were associated with change to schizophrenia: being from London (Nottingham OR 0.34, 95% CI 0.18–0.65); being male (female OR 0.50, 95% CI 0.27–0.92); being in the age risk period (OR 3.46, 95% CI 1.17–10.24); being black African (OR 2.77, 95% CI 1.15–6.65); having a diagnosis of delusional disorder (OR 4.44, 95% CI 0.94–21.00); having symptoms of reality distortion (OR 1.77, 95% CI 1.08–2.91); having negative symptoms (OR 1.77, 95% CI 1.10–2.87); living alone (not living alone OR 0.47, 95% CI 0.25–0.87); being single (not being single OR 0.28, 95% CI 0.13–0.61); not being in further education (further education OR 0.46, 95% CI 0.21–0.99); and having contact with friends less than weekly (OR 3.50, 95% CI 1.24–9.85).

There was strong evidence that the following were associated with not changing diagnosis: having a diagnosis of bipolar disorder (OR 0.13, 95% CI 0.02–0.85); and having manic symptoms (OR 0.37, 95% CI 0.23–0.60). There was some weak evidence that longer DUP was associated with changing diagnosis (OR 1.14, 95% CI 0.98–1.33). For both the ICD and DSM, the numbers were too small to perform multivariate analyses.

## Discussion

The prospective consistencies of both diagnostic systems were comparable at 55–60%. Few demographic, clinical and social factors were associated with overall change in diagnosis but many factors were associated with change to schizophrenia in both DSM and ICD analyses. However, low numbers did not allow for multivariate analysis of variables associated with change to schizophrenia.

### Strengths and limitations

As with most cohort studies, loss to follow-up is a potential bias. Not all incident cases could be given a lifetime diagnosis. However, there was no difference in the proportions followed up between the diagnoses and the overall prevalence of follow-up was respectable at 80%. A further limitation was that missing data in the predictors of change analysis limited the power of the analyses. Despite these limitations, this study contributes evidence beyond previous research because of the incidence sample and 10-year follow-up using both the ICD and DSM diagnoses. Furthermore, it is based on consensus diagnoses made blind to ethnicity and baseline diagnoses and is the first study to examine so many potential predictors of diagnostic change across a range of domains (demographic, clinical and social).

### Findings and implications

An important finding was that schizophrenia, schizo-affective disorder, bipolar disorder and drug-induced psychosis had higher numbers at follow-up, indicating a tendency for other diagnoses to migrate to these categories. This means that the incidence of these diagnoses may be underestimated in incidence studies and this may confuse the aetiological picture.

The prospective consistencies for schizophrenia, bipolar disorder and drug-induced psychosis were significantly higher than the overall stability (>70%). The high stability of drug-induced psychosis is surprising. This category represents a specific subgroup of individuals who demonstrate a clear temporal relationship between onset and recovery of psychotic symptoms and substance use. Rates of co-morbid substance misuse are significant in first-episode psychosis cohorts at around 30–50% (Cantwell *et al.* 1999; Van Mastrigt *et al.* 1999; Barnett *et al.* 2007), whereas drug-induced psychosis often represents less than 10% of a first-episode psychosis cohort (Whitty *et al.* 2005; Addington *et al.* 2006; Bromet *et al.* 2011). This suggests that often drug use is considered by assessors to be co-morbid rather than a sole cause of first-episode psychosis but that where it is identified to be the sole cause, this is often correct.

Depressive psychosis shows lower prospective consistency (45–50%). This might be expected given the anticipation that a substantial percentage of cases will develop bipolar affective disorder over time. However, approximately twice as many cases eventually receive a diagnosis of schizophrenia compared with bipolar disorder. Previous literature is consistent with this finding where consensus diagnosis was used over long periods of follow-up (Bromet *et al.* 2011). It is well recognized that there are prominent symptoms of both anxiety and depression in both prodromal and early psychosis (Birchwood *et al.* 2000) and this is further useful to clinicians considering treatment and prognosis.

The minimal crossover between the bipolar and schizophrenia categories illustrated in the movement matrix is further supported by the finding that a diagnosis of bipolar disorder is associated with reduced odds of changing diagnosis to schizophrenia over time. This taken with the propensity of depressive psychosis cases to change to schizophrenia suggests a complicated relationship between affective and ‘so-called’ non-affective psychosis.

The prospective and retrospective consistencies of the remaining categories were poor (<40%; with the exception of retrospective consistency for delusional disorder). Schizophreniform psychosis greatly overlaps with schizophrenia (duration of symptoms being the only difference) and explains the lower retrospective consistency of the DSM-IV-TR schizophrenia category, perhaps suggesting that it is an unnecessary extra category. Delusional disorder has significantly higher retrospective consistency (50–66%), suggesting less crossover than the other unstable categories. The movement matrix showed more than half of cases with delusional disorder at baseline eventually receive a diagnosis of schizophrenia, and the ICD analysis revealed it is associated with changing diagnosis to schizophrenia. Given this, it may best be considered an attenuated form of schizophrenia.

Examination of the remaining categories in light of their instability – schizo-affective disorder, acute/brief psychosis and psychosis NOS – reveal no immediately obvious patterns or utility in terms of describing a course of symptoms. These categories represent approximately 15% of our sample (ICD 68 cases at baseline, 77 cases at follow-up; DSM-IV 64 cases at baseline, 63 cases at follow-up). This suggests that as well as lacking aetiologically driven diagnoses in psychiatry, for a significant minority of our patients we still lack an adequate descriptive framework, a challenge for new and future revisions of current classification systems.

### Change to diagnosis of schizophrenia

While few characteristics were associated with diagnostic change in general, many factors were associated with change to schizophrenia in both DSM and ICD analyses. Perhaps unsurprisingly, these included variables associated with schizophrenia: symptoms of reality distortion, negative symptoms, and variables indicative of social isolation: living alone, being single and having contact with friends less than weekly. However, low numbers did not allow for multivariate analysis of variables associated with change to schizophrenia. This is in line with previous research that has reported negative symptoms (Mason *et al.* 1997) to be associated with a change in diagnosis to schizophrenia. The finding that social isolation (as measured by contact with friends) was associated with change to schizophrenia may be associated with negative symptoms, as less contact with friends could stem from negative symptoms, and lead to isolation (Schwartz *et al.* 2000).

### Conceptual considerations

The findings from this and other studies on diagnostic stability raise two salient issues. The first is validity: there is an implicit assumption in papers on diagnosis that patients exhibiting stable diagnoses will demonstrate the same as-yet undiscovered physiological abnormalities. The finding that a number of diagnoses change over time is assumed to mean that the initial diagnosis was incorrect and thus the diagnostic systems are flawed, hence research into the causes of these conditions will be hampered. However, in the context of wider medicine, it is a common occurrence for diagnosis to change over time as a disorder develops, or as the clinical picture emerges. It is common that an acute condition precedes a chronic condition, for example, demyelination and multiple sclerosis. In this case, even information on aetiology and mechanism do not indicate outcome, as the mechanism for chronicity is separate. After an initial episode of this neuro-inflammatory disorder, there is little certainty as to prognosis – some people never relapse, for others further episodes occur and the diagnosis becomes multiple sclerosis (McDonald & Compston, 2001). That acute cases of demyelination reoccur and can lead to a diagnosis of multiple sclerosis does not undermine the usefulness of this diagnosis.

This brings us to the second issue of clinical utility. It can be argued that diagnosis informs clinical management of the patient and is therefore useful. However, these results indicate that changes in diagnosis that would probably result in a change of management (e.g. from schizophrenia to bipolar disorder) are minimal, and the changes that do occur are less likely to result in a substantial change in management (e.g. delusional disorder to schizophrenia). Therefore, it could be argued that diagnosis is of little clinical relevance, and specific symptom change is the important thing to be aware of. However, knowledge of how these diagnoses might change over time may prove clinically useful for both doctors and patients in understanding prognosis early in the course of an illness, and, as ever, in quickly communicating the nature of a cluster of symptoms between clinicians.

This raises the question of whether we should be ignoring diagnosis, giving no prognosis to patients and families, and be treating based on symptoms rather than diagnosis. In some early intervention practices, it is now commonplace to assign a broad clinical diagnosis of psychosis early in the course of illness. This avoids using stigmatizing terms such as schizophrenia with its association with poor outcome, promoting the recovery model, and being optimistic about outcomes. It also acknowledges what we have demonstrated in the current study: early on in the course of psychotic illness, the precise diagnosis is often provisional. However, previous research suggests that diagnosis is very strongly associated with outcome (Hegarty *et al.* 1994), and thus not to share this with patients and their families could be unethical. Perhaps a better approach would be to acknowledge the provisional nature of initial diagnosis and be cautious in making inferences regarding prognosis based on diagnosis, as it may not reflect the long-term picture, and to be vigilant for changes in the clinical picture.

The stability of the current classification systems varies widely between diagnoses. Schizophrenia, bipolar disorder, drug-induced psychosis, delusional disorder and depressive psychosis all show relative stability and consistent patterns of change where change does occur. The findings on diagnostic change in depressive and bipolar cases poses a challenge to the distinction of affective/non-affective categories. Psychosis NOS, schizo-affective disorder and acute/brief psychosis perform poorly, yet still provide the best-fit diagnosis for 15% of our cohort, posing a significant challenge in the revision of these categories.

## Supplementary material

For supplementary material accompanying this paper visit http://dx.doi.org/10.1017/S0033291715000720. click here to view supplementary material
