# Supplementary material for: Diagnostic change 10 years after a first episode of psychosis
Source: Psychol Med. 2015 May 4;45(13):2757–69. doi: 10.1017/S0033291715000720 (PMC4595854; doi:10.1017/S0033291715000720)
Supplement: Supplementary file 1 [file S0033291715000720sup001.doc]

Online Supplementary Appendix S1:

Table: Predictors of diagnostic change using the ICD and DSM

| **Predictor** | **ICD Unadjusted OR** | **ICD Final Adjusted OR (n207)** | **Predictor** | **DSM Unadjusted OR** | **DSM Final Adjusted OR (n195)** |
| --- | --- | --- | --- | --- | --- |
| **Demographics** | | | **Demographics** | | |
| **Centre** (n403)  London  Nottingham | -  0.70 (0.46 to 1.04)* | -  0.50 (0.25-1.04)* | **Centre** (n403)  London  Nottingham | -  0.56 (0.38-0.84) ** | -  0.46 (0.22-0.98) ** |
| **Gender** (n403)  Male  Female | -  1.09 (0.73 to 1.63) |  | **Gender** (n403)  Male  Female | -  1.21 (0.82-1.80) |  |
| **Log age** (n403) | 0.96 (0.52 to 1.76) |  | **Log age** (n403) | 0.67 (0.37-1.23) |  |
| **In the age risk period** (n403)  No  Yes | -  1.33 (0.72 to 2.45) |  | **In the age risk period** (n403)  No  Yes | -  1.62 (0.88-2.98) |  |
| **Ethnicity** (n403)  White British  African-Caribbean  Black African  White Other  Asian  Other | -  0.81 (0.49-1.34)  1.52 (0.81-2.88)  0.62 (0.26-1.51)  1.08 (0.45-2.59)  0.60 (0.22-1.63) |  | **Ethnicity** (n403)  White British  African-Caribbean  Black African  White Other  Asian  Other | -  0.81 (0.49-1.32)  1.58 (0.82-3.00)  0.90 (0.39-2.07)  1.13 (0.47-2.68)  0.82 (0.32-2.10) |  |
| **Clinical features** | | | **Clinical features** | | |
| **Diagnosis** (n403)  Schizophrenia  Delusional disorder  Acute and transient Psychoses  Schizoaffective  Bipolar disorder and mania with psychotic features  Major depression with psychotic features  Schizotypal personality disorder  Drug induced psychoses  Psychoses NOS | -  11.34 (3.57-35.96)**  19.14 (5.41-67.79)**  5.37 (2.22-13.02)**  0.94 (0.46-1.90)  3.37 (1.80-6.33)**  -  0.94 (0.33-2.73)  9.07 (3.61-22.77)** | -  23.42 (4.15-132.03)**  73.84 (8.52-639.80)**  9.00 (2.33-34.71)**  1.59 (0.61-4.13)  2.15 (0.77-5.98)  -  3.85 (0.61-24.47)  12.74 (2.24-72.39)** | **Diagnosis** (n403)  Schizophrenia  Major depression with psychotic features  Bipolar disorder and mania with psychotic features  Schizoaffective  Delusional disorder  Psychoses NOS  Schizophreniform  Drug induced psychoses  Other disorder  Unknown/unclear  Brief psychotic disorder | -  3.07 (1.66-5.71)**  0.83 (0.41-1.70)  6.73 (2.45-18.48)**  11.43 (3.64-35.94)**  7.40 (3.06-17.90)**  19.73 (5.61-69.36)**  0.84 (5.61-69.36)  -  -  8.97 (2.35-34.18)** | -  2.54 (0.87-7.41)*  0.97 (0.35-2.74)  12.21 (2.16-69.00)**  40.02 (6.39-250.73)**  22.95 (3.88-135.76)**  -  1.57 (0.23-10.95)  -  -  39.38 (3.53-439.13)** |
| **Family history of psychosis** (n277)  No  Yes | -  0.79 (0.46-1.35) |  | **Family history of psychosis** (n277)  No  Yes | -  0.87 (0.51-1.47) |  |
| **Log DUP days** (n283) | 0.94 (0.85-1.04) |  | **Log DUP days** (n283) | 0.92 (0.83-1.01)* |  |
| **Log age of onset** (n283) | 0.88 (0.48-1.59) |  | **Log age of onset** (n283) | 0.59 (0.33-1.07)* | 0.17 (0.05-0.54)** |
| **Any drug use at baseline** (n367)  No use  Use | -  0.97 (0.64-1.48) |  | **Any drug use at baseline** (n367)  No use  Use | -  1.16 (0.77-1.75) |  |
| **Mode of onset** (n351)  Sudden  Acute  Insidious | -  0.74 (0.39-1.42)  0.78 (0.44-1.36) |  | **Mode of onset** (n351)  Sudden  Acute  Insidious | -  0.84 (0.44-1.58)  0.80 (0.45-1.40) |  |
| Symptom dimension – log mania (n362) | 0.75 (0.56-1.01)* |  | Symptom dimension – log mania (n362) | 0.74 (0.56-0.99)** |  |
| Symptom dimension – log reality distortion (n362) | 0.90 (0.67-1.22) |  | Symptom dimension – log reality distortion (n362) | 1.03 (0.77-1.38) |  |
| Symptom dimension – log negative (n362) | 0.97 (0.71-1.33) |  | Symptom dimension – log negative (n362) | 0.88 (0.64-1.20) |  |
| Symptom dimension – log depression (n362) | 1.55 (1.14-2.11)** | 1.92 (1.11-3.32)** | Symptom dimension – log depression (n362) | 1.62 (1.19-2.20)** | 2.35 (1.30-4.26)** |
| Symptom dimension – log disorganisation (n362) | 0.83 (0.53-1.30) |  | Symptom dimension – log disorganisation (n362) | 0.81 (0.52-1.27) |  |
| **Social Inclusion** | | | **Social Inclusion** | | |
| **Living situation** (n392)  Alone  Not alone | -  0.90 (0.60-1.36) |  | **Living situation** (n392)  Alone  Not alone | -  1.05 (0.70-1.58) |  |
| **Relationship status** (n385)  Single  Not single | -  0.94 (0.59-1.48) |  | **Relationship status** (n385)  Single  Not single | -  0.90 (0.58-1.41) |  |
| **Highest Education level** (n392)  School  Further  Higher | -  0.88 (0.55-1.39)  1.25 (0.65-2.38) |  | **Highest Education level** (n392)  School  Further  Higher | -  0.96 (0.61-1.51)  1.30 (0.68-2.48) |  |
| **Employment status** (n388)  Unemployed  Other | -  1.20 (0.79-1.83) |  | **Employment status** (n388)  Unemployed  Other | -  1.50 (0.99-2.28)* |  |
| **Contact with friends** (n262)  Daily  Weekly  Less than weekly | -  0.98 (0.53-1.79)  0.59 (0.31-1.10)* |  | **Contact with friends** (n262)  Daily  Weekly  Less than weekly | -  0.97 (0.53-1.75)  0.57 (0.31-1.05)* | -  0.61 (0.25-1.49)  0.34 (0.13-0.90)** |

* p<0.10; ** p<0.05
